# Exogenous parathyroid hormone (1–34) attenuates knee osteoarthritis in mice through activating the TGF-β/Smad signaling pathway

**DOI:** 10.1007/s42000-025-00749-w

**Published:** 2026-01-21

**Authors:** Peng Sun, Haoran Jin, Qirui Ding, Haonan Qin, Yunru Ge, Shouguo Wang, Huan Liu

**Affiliations:** 1 Department of Orthopedics, The Affiliated Huaian No.1 People’s Hospital of Nanjing Medical University, Huai’an City, 223300 Jiangsu Province China; 2 Department of Orthopedics, Northern Jiangsu Institute of Clinical Medicine, The Affiliated Huaian No.1 People’s Hospital of Nanjing Medical University, Nanjing Medical University, Huai’an City, 223300 Jiangsu Province China; 3 No. 1, Huanghe West Road, Huaiyin District, Huai’an City, Jiangsu Province 223300 China

**Keywords:** Parathyroid hormone (1–34), Osteoarthritis, TGF-β/Smad signaling pathway, Anterior cruciate ligament transection

## Abstract

**Objective:**

Parathyroid hormone (PTH) has been reported to delay the progression of osteoarthritis (OA), while the mechanism remains unclear. The TGF-β/Smad signaling pathway plays a key role in chondrocyte regulation and OA development. We aimed to explore the effect and mechanism of PTH on knee OA (KOA) in mice.

**Methods:**

Anterior cruciate ligament transection (ACLT) was performed in 40 mice to induce KOA. They were randomly divided into the ACLT + PTH and ACLT + vehicle groups. Another 20 mice that underwent only arthrotomy and normal saline were allocated to the sham-vehicle group. The knee phenotypes were analyzed using radiograph, micro-CT, and histology. The mRNA and protein expression levels of related factors were detected by immunohistochemistry and qRT-PCR.

**Results:**

PTH (1–34) attenuated knee OA phenotypes induced by ACLT, including reducing the modified Mankin’s scores (*p* = 0.002, 0.001) and OARSI scores (*p* = 0.06, 0.02), enhancing type II collagen synthesis (*p* = 0.001, *p* < 0.001), and inhibiting type X collagen (*p* = 0.014, 0.011) and MMP-13 expression (*p* = 0.002, *p* < 0.001) in articular cartilage of ACLT mice at 2 weeks and 4 weeks. PTH also significantly increased the percentage of parathyroid hormone receptor 1 (PTH1R) (*p* < 0.001), p-cAMP response element binding protein (p-CREB) (*p* < 0.001), proliferating cell nuclear antigen (PCNA) (*p* = 0.011, *p* < 0.001), TGF-β receptor I (TβRI) (*p* = 0.002, *p* < 0.001), TβRII (*p* < 0.001), Smad2 (*p* = 0.014, *p* < 0.001), and p-Smad2/3 positive cells (*p* < 0.001) in the articular cartilage of ACLT mice. PTH significantly increased mRNA expression levels of PTH1R, p-CREB, PCNA, TβRI, TβRII, and Smad2 in the articular cartilage of ACLT mice (*p* < 0.001).

**Conclusion:**

PTH (1–34) attentuates knee OA induced by ACLT by stimulating proliferation, inhibiting hypertrophy and maturation of chondrocytes and the degradation of articular cartilage, and activating the TGF-β/Smad signaling pathway.

**Supplementary Information:**

The online version contains supplementary material available at https://doi.org/10.1007/s42000-025-00749-w.

## Introduction

Osteoarthritis (OA) is a prevalent chronic degenerative musculoskeletal disorder characterized clinically by joint pain, tenderness, crepitus, stiffness, and limited mobility,; it is occasionally accompanied by joint effusion and varying degrees of localized inflammation, ultimately leading to functional decline and reduced quality of life [1]. An estimated one-eighth of US adults (27–31 million) suffer from symptomatic OA [2, 3], with projections suggesting 67 million affected individuals by 2030 [4]. The economic burden of OA accounts for approximately 1%–2.5% of GDP in developed countries [5]. OA risk factors are categorized as systemic (age, sex, genetics, and ethnicity) or mechanical (joint alignment, trauma, physical activity, and occupation) [6]. Therapeutic strategies encompass management of modifiable risk factors, intra-articular therapies, physical modalities, and surgical interventions. Early-stage OA treatment primarily targets pain and stiffness relief, while subsequent stages focus on preserving physical function, though no curative approach currently exists.

Articular cartilage defects represent a hallmark pathological feature of OA [7], with chondrocytes serving as key cellular regulators of cartilage homeostasis. These cells constitutively express type II, VI, IX, and XI collagens, cartilage oligomeric matrix protein, and aggrecan to maintain cartilage integrity, bypassing the normal hypertrophic maturation process that leads to matrix mineralization [8]. This contrasts with growth plate chondrocyte behavior during endochondral ossification where cells undergo rounded proliferation, form columnar layers via type II collagen synthesis, and differentiate into prehypertrophic and postmitotic hypertrophic chondrocytes [9]. During OA progression, genes associated with maturation (e.g., type X collagen) and catabolic enzymes (e.g., matrix metalloproteinase-13, MMP-13) exhibit upregulated expression [8]. Additionally, the subchondral bone—comprising the subchondral plate and trabecular bone—plays a pivotal role in OA pathogenesis by supporting overlying articular cartilage and distributing mechanical loads across joint surfaces. Subchondral bone sclerosis may increase load transmission to articular cartilage, thereby promoting cartilage damage [10]. Consequently, suppressing abnormal hypertrophic maturation of chondrocytes, inducing extracellular matrix synthesis, and reducing subchondral bone stiffness represent potential novel therapeutic targets for OA.

Parathyroid hormone (1–34) (teriparatide), the N-terminal 34-amino acid fragment of full-length 84-amino acid PTH secreted by the parathyroid glands, serves as a key calcium homeostasis regulator through direct actions on bone and kidneys and is widely used in osteoporosis treatment [11]. Previous studies demonstrate that PTH (1–34) inhibits terminal differentiation of human articular chondrocytes and attenuates papain-induced rat OA models [12]. In injury-induced murine knee OA models, this peptide exhibits chondroprotective and regenerative effects [13]. PTH (1–34) binds and activates the type I parathyroid hormone receptor (PTH1R), inducing cAMP response element-binding protein (CREB) phosphorylation. Beyond direct chondrocyte actions, one OA model study suggests it may ameliorate synovial pathology [14]. Mendelian randomization analysis (involving 455,221 European participants) revealed an inverse correlation between serum PTH levels and OA risk (particularly knee OA) [15]. Huang et al.‘s Mendelian randomization study further supports a potential causal relationship between reduced circulating PTH and elevated hip/knee OA risk [16].

Transforming growth factor-β (TGF-β) constitutes a pivotal signaling factor regulating chondrogenesis, differentiation, and maturation [17]. TGF-β suppresses chondrocyte hypertrophy and maturation, with its signaling loss leading to cartilage damage, indicating protective role impairment during OA progression [18]. As a downstream effector of the canonical TGF-β/Smad cascade, Smad3 critically influences OA development. Smad3-knockout mice exhibit progressive articular cartilage degeneration resembling human OA [19]. A cohort study of 527 patients demonstrated associations between Smad3 gene single nucleotide polymorphisms (SNPs) and hip/knee OA occurrence rates, reinforcing TGF-β-OA linkages [20]. Another study of 237 knee OA patients and 142 healthy controls identified SMAD3 rs1065080 polymorphism as being correlated with knee OA susceptibility [21]. Analysis of 500 hip OA patients and 1,080 controls confirmed associations between SMAD3 rs1470002/rs12901499 SNPs and hip OA risk [22]. PTH (1–34) has been reported to upregulate TGF-β/Smad signaling across multiple tissues [23]. To investigate whether PTH (1–34) alleviates OA via TGF-β/Smad pathway activation, we employed anterior cruciate ligament transection (ACLT)-induced OA models, administering subcutaneous PTH (1–34) or vehicle postoperatively, then analyzing morphological changes in murine knee joints and TGF-β/Smad pathway molecular expression patterns.

## Materials and methods

### Animal handling

This study was approved by the Institutional Animal Care and Use Committee of Nanjing Medical University. A total of 60 7-week-old C57BL/6 male mice (20–22 g) were purchased from Charles River Laboratories (Beijing, China). They were housed in the Experimental Animal Center at Nanjing Medical University under a 12 h/12 h light/dark cycle. Food and water were provided *ad libitum* in the cages. After 1 week of acclimatization, the mice were randomly assigned to three groups, including the sham-vehicle group, ACLT-vehicle group (the OA induced by ACLT with normal saline), and ACLT-PTH group (the OA with PTH (1–34) treatment) (*n* = 20). Forty mice in the ACLT-vehicle and ACLT-PTH groups underwent ACLT through a medial parapatellar approach. By using a mixture of ketamine (50 mg/kg) and xylazine (5 mg/kg) for anesthesia, the left leg was shaved and disinfected. A medial para-patellar arthrotomy and separation from the medial margin of the quadriceps to the muscles of the medial compartment were performed on the left lower extremity. The patella was laterally dislocated, then the knee joint was fully flexed to expose the articular cavity. Under the direct visualization of the stereomicroscope (NSZ-608T, Novel Optics Co., China), the anterior cruciate ligament was transected and the anterior drawer test was manually performed to further confirm ACLT. The mice in the sham-vehicle group received only arthrotomy. The mice were carefully handled and kept warm during and after surgery. After the experiment, the mice in the ACLT-PTH group were administrated PTH (1–34) (Sigma Chemical Co., USA) at a dosage of 40 µg/kg/d for 5 days per week subcutaneously, while the mice in the sham-vehicle and ACLT-vehicle groups were injected the same volume of normal saline. None of the surgeries failed and no mice died during the experiment. Ten mice in each group were sacrificed at postoperative 2 and 4 weeks, respectively.

### Specimen processing

Half of the operated knee joints were fixed in periodate-lysine-paraformaldehyde (PLP) fixation solution at pH 7.2 to 7.4 for 24 h and then immersed in phosphate buffer solution (PBS) at 4℃ for radiological and histological examinations. The remaining operated knee joints were stored at -80℃ for RNA extraction.

### Radiological examination

Lateral X-ray radiographs were taken using a Faxitron model 805 radiographic inspection system (Faxitron Contact, Faxitron, Germany) (22 kV voltage and 4 min exposure time). Afterwards, the fixed knee joints were analyzed by micro-CT with a SkyScan 1072 scanner and associated analysis software (SkyScan, Antwerp, Belgium). Image acquisition was performed at 100 kV and 98 mA with a voxel size of 9 mm. The region of interest (ROI) was from the distal femur to the proximal tibia. The following morphometric parameters were calculated using software to describe the bone mass and subchondral structure, including bone mineral density (BMD), bone volume ratio (BV/TV), trabecular number (Tb.N), trabecular separation (Tb.Sp), and trabecular thickness (Tb.Th).

### Histological staining and modified mankin’s and OARSI scoring

Knee joints were decalcified in 10% ethylene diamine tetra-acetic acid (EDTA) at 37 °C for 3 weeks, with weekly changes of EDTA, dehydrated in gradient ethanol, embedded in paraffin and serially sectioned at 5 mm in diameter. Hematoxylin & eosin (H&E) staining and safranin-O fast green staining were performed according to standard protocols. Semi-quantitative histopathological analysis was established according to the modified Mankin method [24] and the OA Research Society International (OARSI) microscopic scoring system [25].

### Immunohistochemical assessment

Sections were deparaffinized, hydration, and digested with 0.5% hyaluronidase (Sigma, USA) for 1 h at 37 °C (for collagens) or subjected to antigen retrieval for 5 min at 95 °C (for other indicators). Endogenous peroxidase activity was quenched by incubating the sections with 3% H_2_O_2_. After three washes with PBS, 2% bovine serum albumin was used to block at room temperature for 30 min, and the sections were incubated overnight at 4 °C with the primary antibodies (anti-Collagen II (ab185430, 1:250, Abcam, UK); anti-Collagen X (ab58632, 1:250, Abcam, UK); anti-p-CREB (ab32096, 1:100, Abcam, UK); anti-PTH1R (SAB4502493, 1:250, sigma, USA); anti-PCNA (10205-2-AP,1:250, Proteintech, USA); anti-TβRI (ab31013,1:50, Abcam, UK); anti-TβRII (ab186838, 1:250, Abcam, UK); anti-Smad2 (ab63576, 1:150, Abcam, UK); anti-p-Smad2/3 (ab663399, 1:150, Abcam, UK), and anti-MMP13 (ab39012, 1:250, Abcam, UK)) followed by three washes with PBS. Goat anti-mouse IgG (FAB segment) antibody or goat anti-rabbit antibody (Roche, Switzerland) was incubated for 1 h at room temperature. After three washes with PBS, the Vectastain Elite ABC kit (Vector, USA) was added for 30 min at room temperature. Diaminobenzidine (DAB) Perooxidase Substrate Kit (Vector, USA) was used as a peroxidase substrate. The sections were counterstained with hematoxylin and mounted with a neutral resin solution. Images were captured on a NIKON E600 Microscope with a SPOT2 digital camera using SPOTcam v.3.5.5 software (Digital Instruments, Inc., Buffalo, NY, USA).

### RNA extraction and real-time quantitative reverse transcription polymerase chain reaction (qRT-PCR)

Total RNA was collected from articular cartilage by using TRIzol reagent (Beyotime, Shanghai, China). Subsequently, RNA was applied to reverse transcription to cDNA by using HiScript III Reverse Transcriptase (Vazyme, Nanjing, China). qRT-PCR was performed by using the ABI 7500 Fast Real-Time PCR System (Applied Biosystems). Target gene primers included Collagen II (GGGAATGTCCTCTGCGATGAC (forward) and GAAGGGGATCTCGGGGTTG (reverse)), Collagen X (TTCTGCTGCTAATGTTCTTGACC (forward) and GGGATG AAGTATTGTGTCTTGGG (reverse)), MMP-13 (CTTCTTCTTGTTGAGCTGGAC TC (forward) and CTGTGGAGGTCACTGTAGACT (reverse)), CREB (ACCCAGG GAGGAGCAATACAG (forward) and TGGGGAGGACGCCATAACA (reverse)), PCNA (TTTGAGGCACGCCTGATCC (forward) and GGAGACGTGAGACGAG TCCAT (reverse)), TβRI (TCTGCATTGCACTTATGCTGA (forward) and AAAGGGCGATCTAGTGATGGA (reverse)), TβRII (CCGCTGCATATCGTCCT GTG (forward) and AGTGGATGGATGGTCCTATTACA (reverse)), Smad2 (ATGT CGTCCATCTTGCCATTC (forward) and AACCGTCCTGTTTTCTTTAGCTT (reverse)), and GADPH (ATGATTCTACCCACGGCAAG (forward) and CTGGAAG ATGGTGATGGGTT (reverse)). Expression levels of target genes were normalized to GAPDH by using the 2^−ΔΔCt^ method.

### Statistical analyses

SPSS 26.0 software package (SPSS Inc., Chicago, IL, USA) was used for statistical analyses. Numerical data are presented as means ± standard deviation (SD). Student’s *t*-test was performed to evaluate the differences among groups. Significances were considered statistically significant at *P*<0.05.

## Results

### PTH attenuated ACLT-induced knee OA

**Fig. 1:**
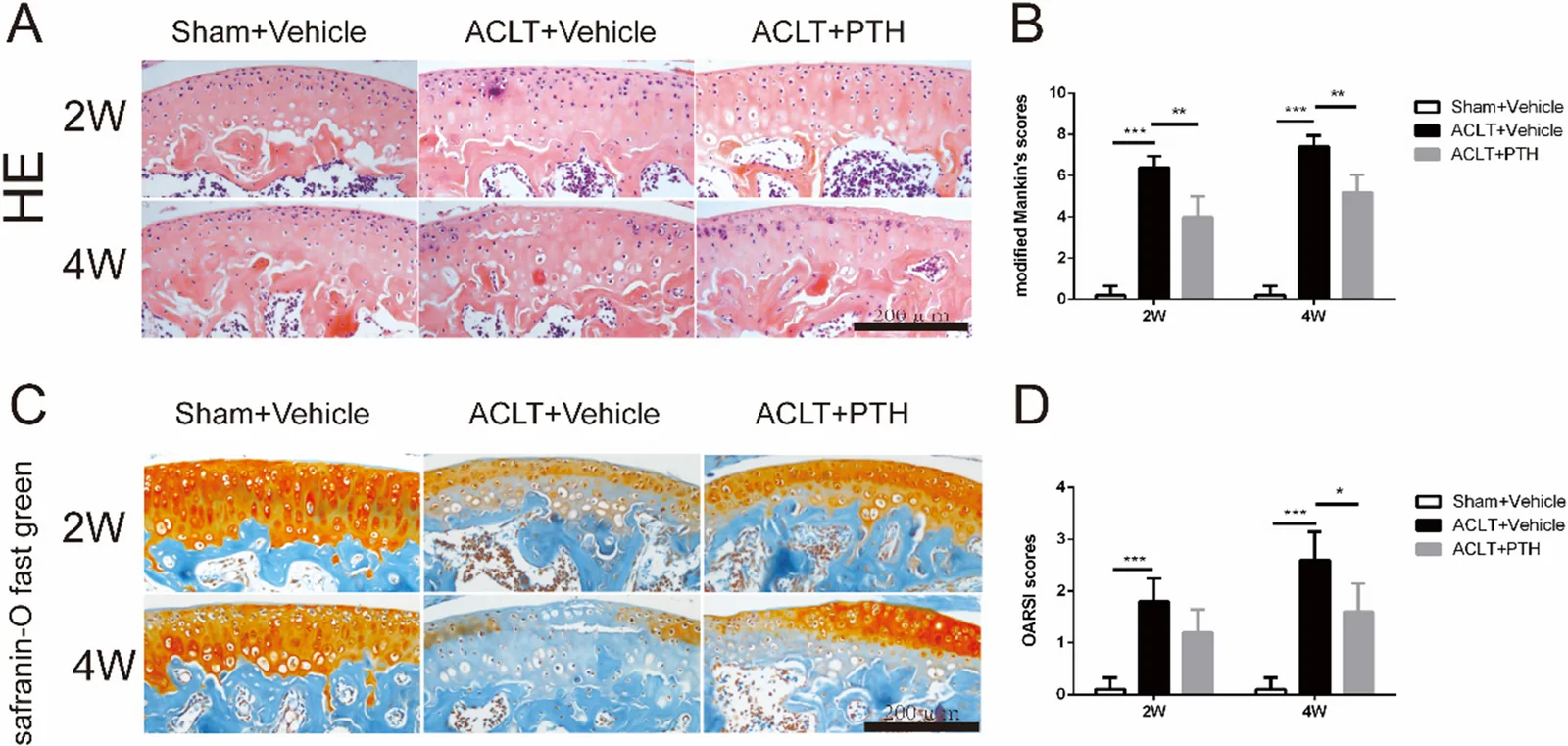
PTH attenuated ACLT-induced knee OA. (**A**) H&E staining; (**B**) modified Mankin staining; (**C**) safranin-O fast green staining; (**D**) OARSI scores of the articular cartilage. ACLT, anterior cruciate ligament transection. Quantitative variables are presented as mean ± standard deviation; *n* = 3. Compared with ACLT + vehicle group, ^*^P < 0.05, ^**^P < 0.01, ^***^P < 0.001. Magnification, 100×

To investigate whether PTH alleviates ACLT-induced knee OA, mice were subcutaneously administered either vehicle or PTH (1–34) for 2 or 4 weeks post-ACLT. Phenotypic analysis of knee joints revealed that ACLT caused disorganized chondrocyte distribution, reduced cell numbers, and blurred or even absent tidemark in articular cartilage. Compared with the sham group, the ACLT-vehicle group exhibited significantly higher modified Mankin scores (*p* < 0.001) and OARSI scores (*p* < 0.001), whereas PTH administration partially reversed these parameters (*p* = 0.002, 0.001, 0.007, and 0.024, Fig. 1A-D). Detailed comparative results are presented in Supplementary Table 1. These results indicate that PTH (1–34) mitigates ACLT-induced knee OA.

### PTH rescued subchondral bone loss induced by ACLT

**Fig. 2:**
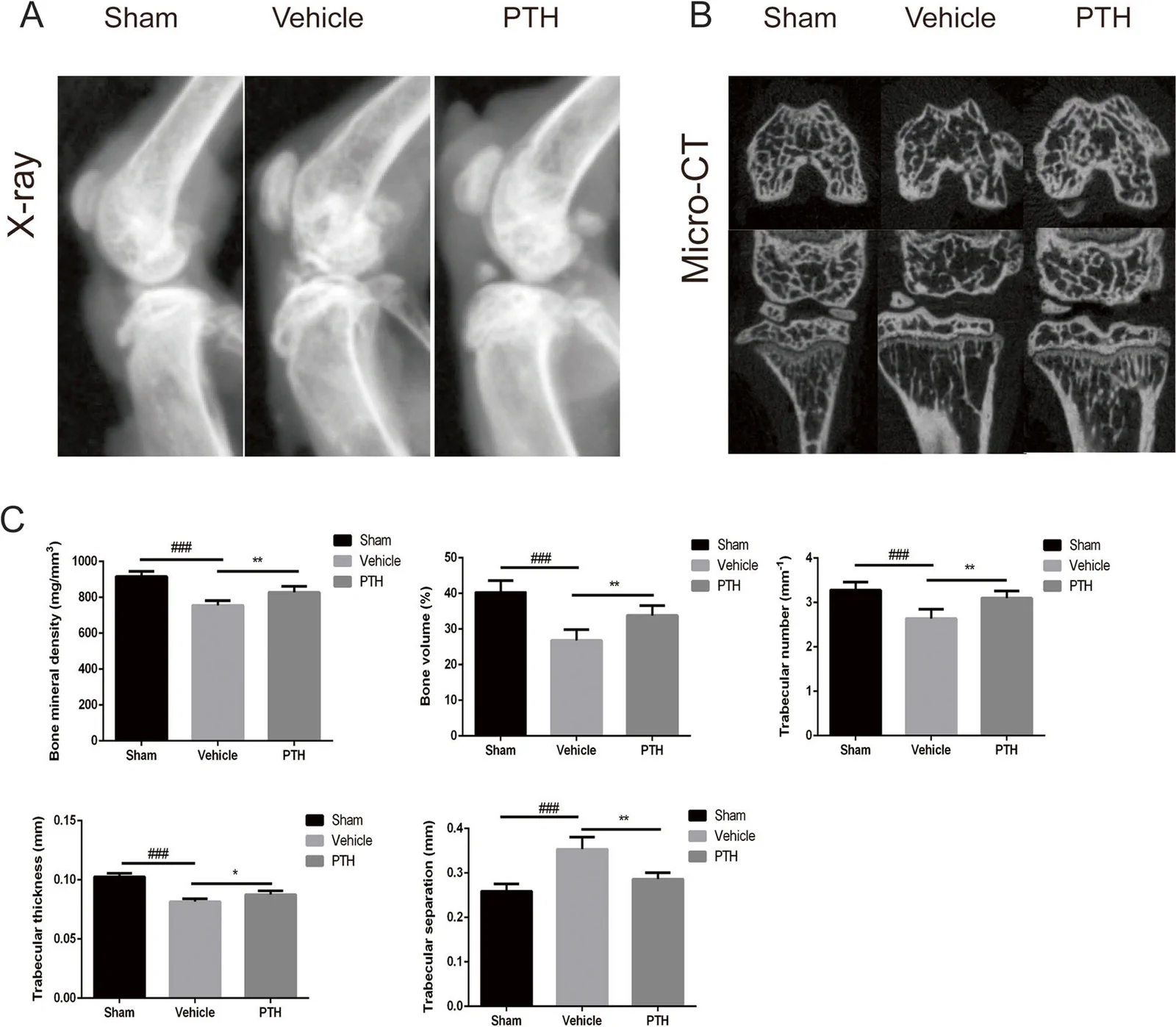
PTH rescued subchondral bone loss induced by ACLT. Transversal and coronal reconstruction images (**A**), and parameters (**B**) of micro-CT scans. (**C**) the levels of bone mineral density, bone volume (%), trabecular number, trabecular separation, and trabecular thickness. Quantitative variables are presented as mean ± standard deviation; *n* = 3. Compared with ACLT + vehicle group, ^*^P < 0.05, ^**^P < 0.01; compared with sham group, ^###^*P* < 0.001

The effect of PTH on subchondral bone loss was evaluated by radiography and micro-CT analysis at 4 weeks post-surgery. Compared with the sham group, the ACLT-vehicle group showed significantly reduced BMD, trabecular bone volume, trabecular number, and trabecular thickness, along with increased trabecular separation (*p* < 0.001). PTH treatment significantly improved these parameters (*p* = 0.004, *p* = 0.003, *p* = 0.005, *p* = 0.041, *p* = 0.004) (Fig. 2A-C). Detailed comparative results are presented in Supplementary Table 1. These findings suggest that PTH effectively rescues ACLT-induced subchondral bone loss.

### PTH enhanced type II collagen but reduced type X collagen and MMP-13 expression levels in articular cartilage of ACLT mice

**Fig. 3:**
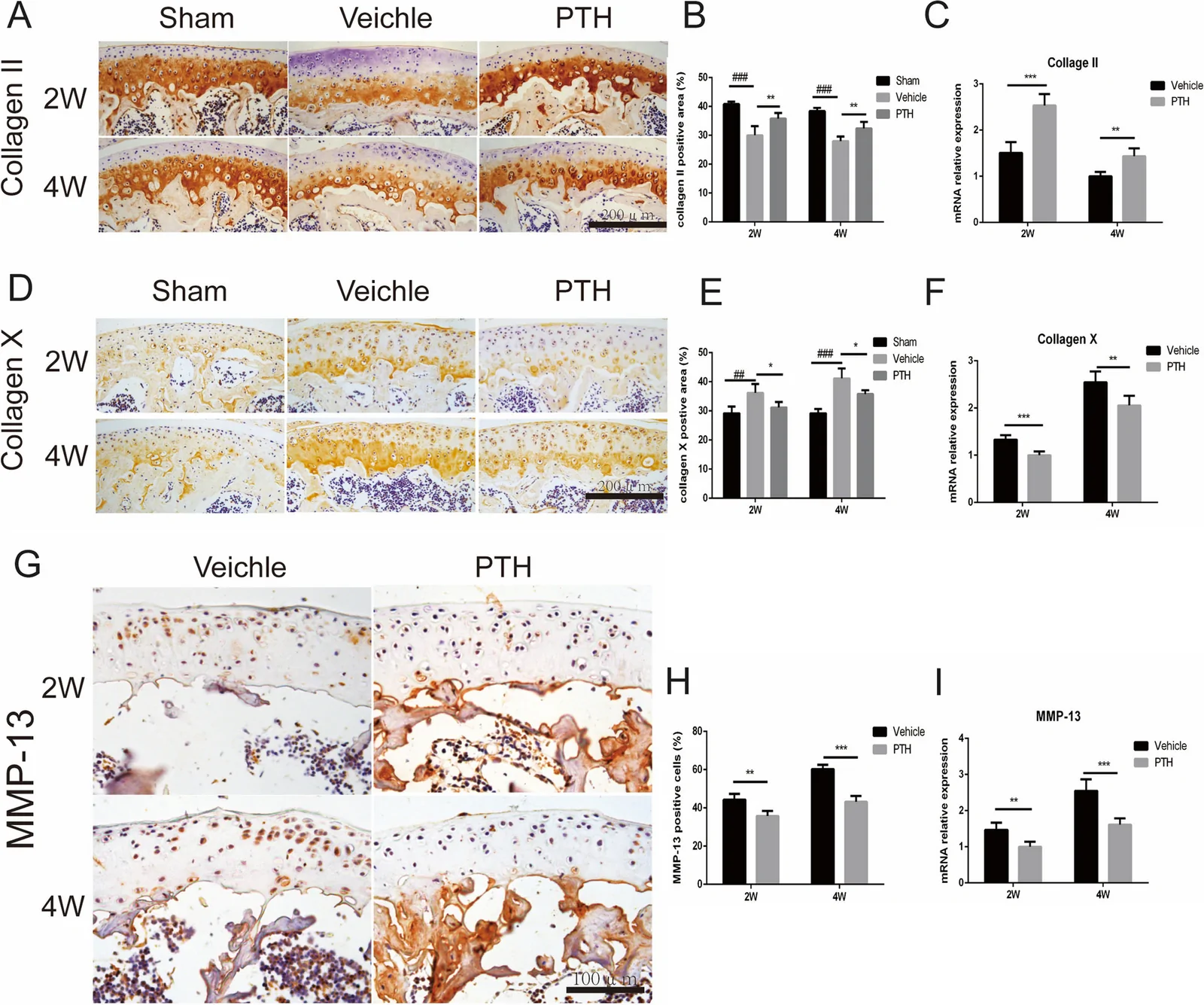
PTH enhanced type II collagen expression levels but reduced type X collagen and MMP-13 expression levels in the articular cartilage of ACLT mice. Protein (**A**, **B**) and mRNA levels (**C**) of type II collagen in articular cartilage at 2 and 4 weeks after surgery. Protein (**D**, **E**) and mRNA expression levels (**F**) of type X collagen in articular cartilage at 2 and 4 weeks after surgery. Protein (**G**, **H**) and mRNA expression levels (I) of MMP-13 in articular cartilage at 2 and 4 weeks after surgery. A and D: magnification 100×. G: magnification, 200×. Quantitative variables are presented as mean ± standard deviation; *n* = 3. Compared with ACLT + vehicle group, ^*^P < 0.05, ^**^P < 0.01, ^***^P < 0.001; compared with sham group, ^##^*P* < 0.01, ^###^*P* < 0.001

Immunohistochemistry and qRT-PCR were performed to assess the expression levels of type II collagen, type X collagen, and MMP-13 in articular cartilage at 2 and 4 weeks post-surgery. Compared with the sham group, the ACLT-vehicle group exhibited significantly decreased type II collagen-positive area (*p* < 0.001), whereas type X collagen-positive areas (*p* = 0.003, *p* < 0.001) (*p* < 0.001, *p* = 0.004) were markedly elevated (Fig. 3). PTH administration significantly increased type II collagen-positive areas (*p* = 0.003, *p* = 0.007) and mRNA expression (*p* < 0.001, *p* = 0.002) and decreased type X collagen-positive areas (*p* = 0.043, *p* = 0.021) and mRNA expression (*p* < 0.001, *p* = 0.007) and MMP-13 -positive areas (*p* = 0.006, *p* < 0.001) and mRNA expression (*p* = 0.004, *p* < 0.001) (Fig. 3A-I). Detailed comparative results are presented in Supplementary Table 1. These results indicate that PTH alleviates ACLT-induced knee osteoarthritis by upregulating type II collagen and downregulating type X collagen and MMP-13 expression.

### PTH stimulated chondrocyte proliferation mediated via PTH1R in articular cartilage of ACLT mice

**Fig. 4:**
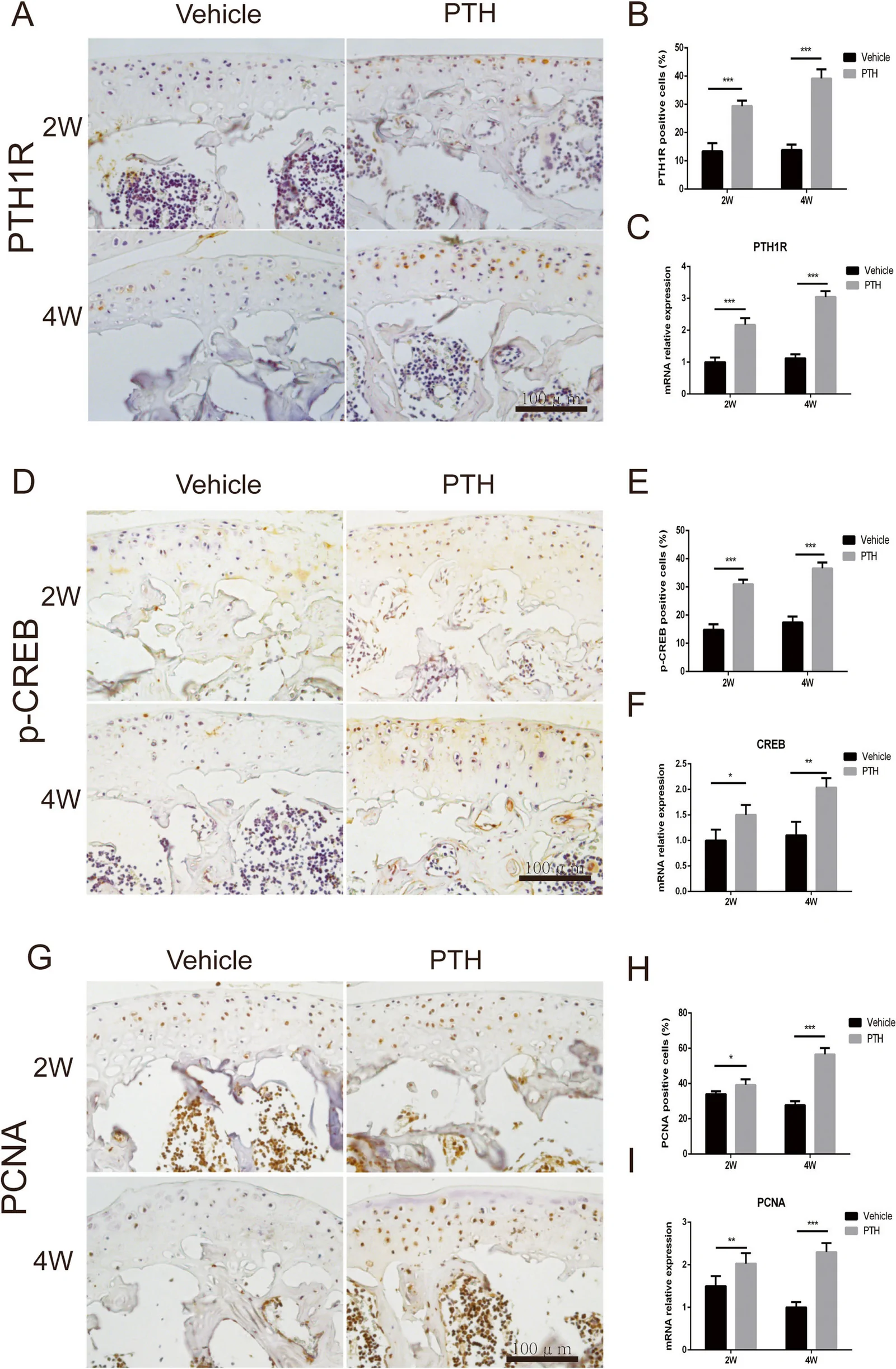
PTH stimulated chondrocyte proliferation mediated via PTH1R in articular cartilage of ACLT mice. Protein (**A**, **B**) and mRNA (**C**) expression levels of PTHIR in articular cartilage at 2 and 4 weeks after surgery. Protein expression level of p-CREB (**D**, **E**) and mRNA expression level of CREB (**F**) in articular cartilage at 2 and 4 weeks after surgery. Protein (**G**, **H**) and mRNA (**I**) expression levels of PCNA in articular cartilage at 2 and 4 weeks after surgery. Quantitative variables are presented as mean ± standard deviation; *n* = 3. Compared with ACLT + vehicle group, ^*^P < 0.05, ^**^P < 0.01, ^***^P < 0.001. Magnification, 200×

The expression of PTH1R, p-CREB, and PCNA was examined. Compared with the ACLT-vehicle group, PTH-treated mice at 2 and 4 weeks showed significantly increased proportions of PTH1R-, p-CREB-, and PCNA-positive cells (*p* < 0.001, *p* = 0.024), along with elevated mRNA levels (*p* < 0.001, *p* = 0.035, 0.004, 0.009), with more pronounced effects at 4 weeks (Fig. 4A-I). Detailed comparative results are presented in Supplementary Table 2. These findings suggest that PTH mitigates ACLT-induced knee osteoarthritis by promoting chondrocyte proliferation via PTH1R-mediated signaling.

### PTH activated TGF-β/Smad signaling pathway in articular cartilage of ACLT mice

**Fig. 5:**
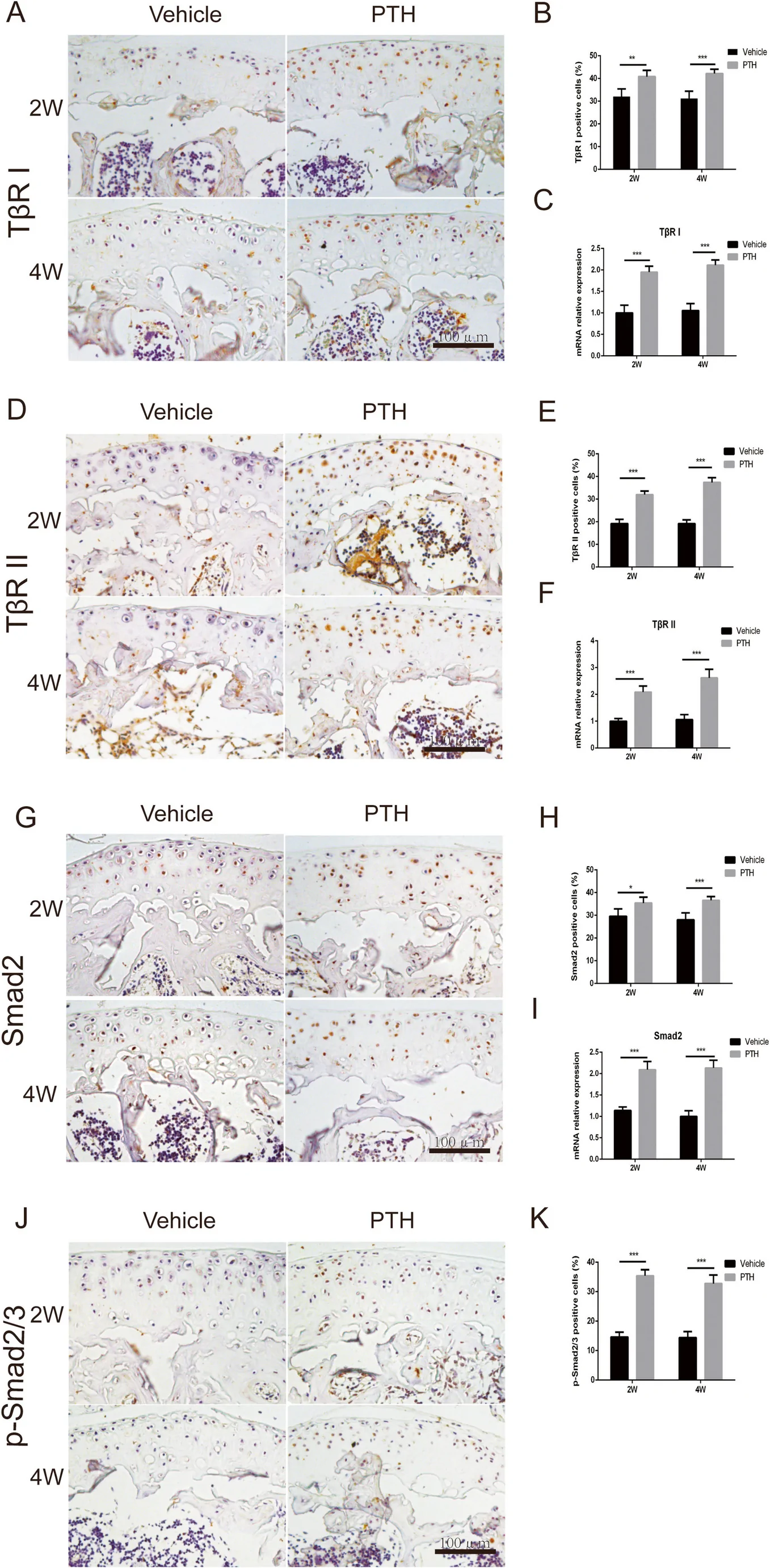
PTH activated the TGF-β/Smad pathway in articular cartilage of ACLT mice. Protein (**A**, **B**) and mRNA (**C**) expression levels of TβR I in articular cartilage at 2 and 4 weeks after surgery. Protein (**D**, **E**) and mRNA (**F**) expression levels of TβR II in articular cartilage at 2 and 4 weeks after surgery. Protein (**G**, **H**) and mRNA (**I**) expression levels of Smad2 in articular cartilage at 2 and 4 weeks after surgery. Protein expression levels of p-Smad2/3 in articular cartilage at 2 and 4 weeks after surgery (**J**, **K**). Quantitative variables are presented as mean ± standard deviation; *n* = 3. Compared with ACLT + vehicle group, ^*^P < 0.05, ^**^P < 0.01, ^***^P < 0.001. Magnification, 200×

Analysis of TGF-β signaling-related factors, including TβRI, TβRII, Smad2, and p-Smad2/3, revealed that PTH treatment significantly increased the proportions of TβRI- (*p* = 0.002, *p* < 0.001), TβRII- (*p* < 0.001), Smad2- (*p* = 0.014, *p* = 0.001), and p-Smad2/3-positive cells (*p* < 0.001), as well as the mRNA levels of TβRI (*p* < 0.001), TβRII (*p* < 0.001), and Smad2 (*p* < 0.001), compared with the ACLT-vehicle group (Fig. 5A-K). Detailed comparative results are presented in Supplementary Table 2. These results demonstrate that PTH alleviates ACLT-induced knee osteoarthritis by activating the TGF-β/Smad signaling pathway in articular cartilage.

## Discussion

This study is the first to untangle the complex mechanism by which PTH(1–34) exerts therapeutic effects on OA through PTH1R-mediated activation of the TGF-β/Smad signaling pathway. We demonstrate for the first time that PTH(1–34) enhances TGF-β signaling sensitivity by upregulating the expression of TβRI and TβRII while simultaneously promoting Smad2 transcription and Smad2/3 phosphorylation, thereby establishing a complete signaling cascade. Notably, this regulatory role concurrently ameliorates subchondral bone loss and articular cartilage degeneration, representing a previously unreported dual protective effect. Furthermore, we elucidate the manner in which PTH(1–34)-induced chondrocyte proliferation through p-CREB6 and PCNA1 exhibits synergistic effects with Smad pathway activation. This crosstalk regulatory network presents significant novelty in the field of OA research. In contrast to previous studies focusing solely on PTH’s impact on individual tissues or pathways, this work systematically reveals the comprehensive regulatory role of the PTH(1–34)-PTH1R-TGF-β/Smad axis in the osteochondral unit.

The ACLT-induced OA model used in this study is a classic post-traumatic OA (PTOA) animal model [26, 27]. In our study, from 2 to 4 weeks after ACLT, the articular cartilage became progressively impaired with thinner articular cartilage thickness, disordered chondrocyte distribution, and decreased chondrocyte number. The boundary between the non-calcified layer and the calcified layer of articular cartilage is called the tidemark. An intact tidemark is a sign of normal articular cartilage. We found that the tidemark became vague or even disappeared in histological examinations of the ACLT-vehicle mice. The modified Mankin and OARSI scores, reflecting articular cartilage morphology, were significantly increased in ACLT-vehicle mice when compared to those of the sham mice. At the cellular level, inappropriate differentiation was related to the up-regulation of type X collagen and MMP-13, assembly of inflammation indicators, and aberrant maturation of articular chondrocytes which contributed to OA progression [7, 28].

PTH (1–34) has been reported to trigger chondroprotective and chondro-regenerative effects [15], suppress the terminal differentiation of human articular chondrocytes [29], and reduce synoviopathy [16]. Herein, we demonstrated that PTH (1–34) administration was able to decrease modified Mankin and OARSI scores by maintaining cartilage structure. PTH binds to PTH1R in order to phosphorylate CREB to activate PCNA and promote chondrocyte proliferation. Moreover, it inhibits chondrocyte hypertrophy and differentiation in normal cartilage development [30]. There were upregulated expressions of PTH1R, p-CREB, and PCNA triggered by PTH (1–34) in this study. In MSCs from OA patients, PTH (1–34) inhibited the expression of type X collagen in a time-dependent manner and stimulated the expression of type II collagen [31]. Dexamethasone (Dex) was able to induce terminal differentiation of articular chondrocytes, while it was observed that PTH (1–34) treatment reverses both changes of chondrogenic and hypertrophic markers, decreases Dex-induced cell death, and reduces Dex-induced terminal differentiation and apoptosis of articular chondrocytes to protect articular cartilage from further damage [32]. In an avian sternal organ culture model, PTH (1–34) increased cartilaginous tissue length and downregulated the deposition of type X collagen and its mRNA expression. Similar alterations were detected in our study. Following 4-week administration of PTH (1–34), type II collagen protein and mRNA expression were increased but type X collagen was decreased. MMP13 which induces chondrocyte differentiation was suppressed by the administration of PTH (1–34). It also raised chondrocyte cell diameter in pre-hypertrophic and proliferative regions while inhibiting chondrocyte apoptosis in the hypertrophic zone. Furthermore, in a previous study designed to investigate the repair of articular cartilage injury in rabbits, the loss of articular cartilage was filled with hyaline tissue by using PTH-induced bone marrow mesenchymal stem cells (BM-MSCs) complexed with fibrin glue at 12 weeks after surgery. Typical cartilage lacunae, intact subchondral bone, and upregulated expression of type II collagen and aggrecan were found [33].

PTH(1–34) is primarily recognized as a regulator of calcium homeostasis, achieved through its stimulatory effect on bone remodeling. This is accomplished via direct action on osteoblasts and osteocytes, while its indirect influence on osteoclasts is mediated through its effects on osteoblastic bone cells and skeletal cells [34]. PTH(1–34) stimulates both bone resorption and bone formation, and the ultimate result on bone mass, whether catabolic or anabolic, is dependent on the dosage and administration schedule of PTH(1–34). Continuous exposure to PTH(1–34) exerts a catabolic effect on the skeleton, whereas intermittent, low-dose administration of PTH(1–34) induces an anabolic effect on bone [35]. In joints, PTH(1–34) can delay the deterioration of subchondral trabecular bone, thereby preventing the progression of cartilage damage in guinea pigs with spontaneous osteoarthritis [36]. In this study, intermittent low-dose injections of PTH(1–34) were employed, leading to increases in BMD, BV/TV, Tb.N, and Tb.Th, but a decrease in Tb.Sp. The structure of the subchondral bone was well maintained to support the articular surface.

The TGF-β superfamily represents a group of multifunctional growth factors that play important roles in both physiological and pathological conditions [37–39]. TGF-β can bind to TβRII, activating the canonical TGF-β/Smad signaling cascade. In BM-MSCs, PTH(1–34) coordinates and activates matrix TGF-β, and dysregulation of TGF-β alters the fate of BM-MSCs, decoupling bone remodeling and leading to skeletal diseases [21]. In cultured human osteoblasts, PTH(1–34) increases the concentration and gene transcription of TGF-β [22]. In degenerative disc disease, PTH(1–34) induces the transcription of integrin αγβ6 to activate the TGF-β-connective tissue growth factor (CCN2)-matrix protein signaling cascade. Intermittent injection of PTH(1–34) effectively attenuates disc degeneration in aged mice through direct signaling in nucleus pulposus cells, specifically improving disc height and volume by enhancing TGF-β activity, CCN2, and aggrecan levels [23]. The differentiation and maturation of chondrocytes are tightly regulated by several key growth factors [40] and transcription factors, including members of the TGF-β superfamily [17]. However, whether PTH(1–34) alleviates OA through the TGF-β/Smad signaling pathway remains unclear.

In this study, we discovered that the expression of TβRII, TβRI, Smad2, and p-Smad2/3 was triggered by the administration of PTH (1–34). In the canonical pathway, TGF-β binds to TβRII, which then phosphorylates the transmembrane serine/threonine kinase of TβRI. Subsequently, TβRI phosphorylates Smads 2 and 3 (R-Smad) at their C-termini. As a result, the activated R-Smads dissociate from the receptor complex and form a heterodimeric complex with the common Smad4. This heterodimeric Smad complex then enters the nucleus and binds to other DNA-binding proteins to regulate target gene transcription [41]. Primary rabbit condylar chondrocytes treated with TGF-β1 showed increased synthesis of type II collagen and cell growth [42]. In cultured rat chondrocytes, TGF-β1 stimulates cell proliferation and extracellular matrix formation through the MEK-ERK-Elk1 pathway [43]. TGF-β helps maintain the matrix components of cartilage in an immature state [44, 45]. Through the Smad-dependent pathway, TGF-β induces the expression of aggrecan in chondrocyte lineages. Under the action of TGF-β, Smad2 is rapidly phosphorylated, leading to the initial activation of the aggrecan gene. Recent genetic manipulations of TGF-β signaling members, including the overexpression of dominant-negative TβR II in skeletal tissue [46], conditional knockout of TβRII in chondrocytes [47], and deletion of Smad3 in chondrocytes [43], also suggest that TGF-β signaling plays a key role in the development of OA. Therefore, we hypothesize that PTH (1–34) may activate the TGF-β/Smad signaling pathway to mitigate the progression of OA.

There are some limitations in this study. The experiment was performed in vivo merely, lacking in vitro results to for confirmation. The time-series detection and comparison finished at 4 weeks after ACLT. Long-term and more comprehensive investigations would be considered in further research.

## Conclusion

The results of this study demonstrate that PTH effectively alleviates knee OA induced by ACLT. PTH(1–34) treatment significantly ameliorated ACLT-induced structural cartilage damage and subchondral bone loss while maintaining cartilage homeostasis through upregulating type II collagen expression and downregulating type X collagen and MMP-13 expression. Mechanistically, PTH activated both CREB and TGF-β/Smad signaling pathways via PTH1R, thereby promoting chondrocyte proliferation and inhibiting degenerative changes. These findings provide a theoretical foundation for PTH as a potential therapeutic strategy for osteoarthritis.

## Supplementary Information

Below is the link to the electronic supplementary material.

Supplementary Material 1

## Data Availability

The simulation experiment data used to support the findings of this study are available from the corresponding author upon request.
