# Supplementary material for: Exogenous parathyroid hormone (1–34) attenuates knee osteoarthritis in mice through activating the TGF-β/Smad signaling pathway
Source: Hormones (Athens). 2026 Jan 21;25(2):417–28. doi: 10.1007/s42000-025-00749-w (PMC13582118; doi:10.1007/s42000-025-00749-w)
Supplement: Supplementary file 1 [file 42000_2025_749_MOESM1_ESM.docx]

**Supplementary Table 1. Summary of Key Quantitative Results at 2 and 4 Weeks Post-ACLT.**

| Parameters | Time Point (Week) | Sham+Vehicle | ACLT+Vehicle | ACLT+PTH | p-value (ACLT+Vehicle vs Sham+Vehicle) | p-value (ACLT+Vehicle vs ACLT+PTH) |
| --- | --- | --- | --- | --- | --- | --- |
| Histological Scores | | | | | | |
| Mankin's Score | 2 | 0.2 ± 0.447 | 6.4± 0.548 | 4.0± 1.000 | < 0.0001 | 0.001528 |
|  | 4 | 0.2 ± 0.447 | 7.4± 0.548 | 5.2 ± 0.837 | < 0.0001 | 0.001165 |
| OARSI Score | 2 | 0.2 ±0.447 | 1.8± 0.447 | 1.2 ± 0.447 | < 0.0001 | 0.06669 |
|  | 4 | 0.2 ±0.447 | 2.6 ± 0.548 | 1.6 ± 0.548 | < 0.0001 | 0.020300 |
| **Positive Cells (IHC)** | | | | | | |
| Collagen II | 2 | 85.80± 4.60 | 54.20± 4.60 | 67.40± 3.05 | < 0.0001 | 0.000690 |
|  | 4 | 86.80± 5.40 | 28.40± 4.83 | 51.40 ± 4.04 | < 0.0001 | < 0.0001 |
| Collagen X | 2 | 29.20± 2.28 | 36.20± 3.03 | 31.20± 1.92 | 0.003322 | 0.01438 |
|  | 4 | 29.20 ± 1.48 | 41.20 ± 3.42 | 35.80± 1.30 | < 0.0001 | 0.01089 |
| MMP-13 | 2 | 31.80± 3.03 | 44.20 ± 3.19 | 35.80 ± 2.59 | 0.0002340 | 0.001828 |
|  | 4 | 31.20 ± 3.19 | 60.20 ± 2.28 | 43.20 ± 3.03 | < 0.0001 | < 0.0001 |
| mRNA Expression**%** | | | | | | |
| Collagen II | 2 | 5.83±0.37 | 1.51±0.23 | 2.53±0.25 | < 0.0001 | < 0.0001 |
|  | 4 | 5.78±0.30 | 1.00±0.10 | 1.44±0.17 | < 0.0001 | 0.001243 |
| Collagen X | 2 | 1.00±0.08 | 2.69±0.08 | 2.06±0.20 | < 0.0001 | 0.000133 |
|  | 4 | 1.00±0.20 | 3.55±0.31 | 2.58±0.28 | < 0.0001 | 0.000966 |
| MMP-13 | 2 | 1.00±0.14 | 3.47±0.20 | 2.69±0.30 | < 0.0001 | 0.000983 |
|  | 4 | 1.04±0.20 | 5.02±0.26 | 3.61±0.17 | < 0.0001 | < 0.0001 |
| Micro-CT Parameters | | | | | | |
| BMD | 4 | 914.40±29.211 | 755.20± 27.28 | 827.60± 33.26 | < 0.0001 | 0.005518 |
| BV/TV | 4 | 40.40± 3.21 | 26.80± 3.03 | 33.80±2.77 | 0.0001262 | 0.005181 |
| Tb.N | 4 | 3.28± 0.18 | 2.64±0.21 | 3.1±0.16 | 0.0007972 | 0.004269 |
| Tb.Th | 4 | 0.10±0.00 | 0.08±0.00 | 0.09±0.00 | < 0.0001 | 0.01018 |
| Tb.Sp | 4 | 0.26±0.02 | 0.35±0.03 | 0.29±0.01 | 0.0001488 | 0.001142 |

Data presented as Mean ± Standard Deviation

**Supplementary Table 2. Summary of Key Quantitative Results at 2 and 4 Weeks Post-ACLT**

| Parameter | Time Point | ACLT+Vehicle | ACLT+PTH | p-value |
| --- | --- | --- | --- | --- |
| **% Positive Cells (IHC)** | | | | |
| PTH1R | 2 Weeks | 13.40± 2.88 | 29.40± 1.95 | < 0.0001 |
|  | 4 Weeks | 13.80± 1.92 | 39.20 ± 3.19 | < 0.0001 |
| CREB | 2 Weeks | 14.80± 1.92 | 31.00 ± 1.58 | < 0.0001 |
|  | 4 Weeks | 17.40 ± 2.07 | 36.60± 2.07 | < 0.0001 |
| TβRI | 2 Weeks | 32.00± 3.39 | 40.80± 2.77 | 0.002027 |
|  | 4 Weeks | 31.20± 3.19 | 42.20± 1.92 | 0.000170 |
| TβRII | 2 Weeks | 19.20±1.92 | 32.00±1.58 | < 0.0001 |
|  | 4 Weeks | 19.20± 1.64 | 37.40±2.07 | < 0.0001 |
| Smad2/3 | 2 Weeks | 29.60±3.21 | 35.40± 2.61 | 0.013883 |
|  | 4 Weeks | 28.00± 3.08 | 36.60± 1.67 | 0.0001 |
| PCNA | 2 Weeks | 34.00± 1.58 | 39.20±3.19 | 0.000585 |
|  | 4 Weeks | 27.80± 2.17 | 56.60±3.51 | < 0.0001 |
| p-Smad2/3 | 2 Weeks | 14.60± 1.67 | 35.40± 2.07 | < 0.0001 |
|  | 4 Weeks | 14.40± 2.07 | 32.80± 2.86 | < 0.0001 |
| mRNA Expression | | | | |
| PTH1R | 2Weeks | 1.00±0.15 | 2.18±0.20 | < 0.0001 |
|  | 4Weeks | 1.12±0.13 | 3.04±0.18 | < 0.0001 |
| PCNA | 2Weeks | 1.50±0.23 | 2.03±0.25 | 0.007823 |
|  | 4Weeks | 1.00±0.13 | 2.30±0.21 | < 0.0001 |
| TβRI | 2 | 1.00±0.18 | 1.95±0.14 | < 0.0001 |
|  | 4 | 1.05±0.16 | 2.11±0.12 | < 0.0001 |
| Smad2/3 | 2Weeks | 1.14±0.08 | 2.09±0.19 | < 0.0001 |
|  | 4Weeks | 1.00±0.13 | 2.13±0.18 | < 0.0001 |
| CREB | 2Weeks | 1.00±0.21 | 1.50±0.19 | 0.04018 |
|  | 4Weeks | 1.10±0.27 | 2.04±0.18 | 0.007012 |

Data presented as Mean ± Standard Deviation
